# Post-hurricane fluid conservation measures fail to reduce IV fluid use in critically ill children

**DOI:** 10.1007/s00467-025-06931-x

**Published:** 2025-08-19

**Authors:** Celeste G. Dixon, James D. Odum, Ulka Kothari, Susan D. Martin, Julie C. Fitzgerald, Ami Shah, Heda Dapul, Chloe G. Braun, Andrew Barbera, Nina Terry, Scott L. Weiss, Denise C. Hasson, Adam C. Dziorny

**Affiliations:** 1 Dept of Anesthesiology & Critical Care Medicine, Children’s Hospital of Philadelphia, The University of Pennsylvania Perelman School of Medicine, Philadelphia, PA USA; 2 Dept of Pediatrics, University of Alabama at Birmingham, Birmingham, AL USA; 3 Dept of Pediatrics, NYU Long Island School of Medicine, Mineola, NY USA; 4 Dept of Pediatrics, Golisano’s Children’s Hospital, University of Rochester School of Medicine, Rochester, NY USA; 5 Dept of Pediatrics, Hassenfeld Children’s Hospital at NYU Langone Health, New York, NY USA; 6 Division of Critical Care, Dept of Pediatrics, Nemour’s Children’s Hospital, Wilmington, DE USA

**Keywords:** Pediatric critical care, Fluid balance, Intravenous infusion, Disaster management

## Abstract

**Background:**

There are risks associated with excessive intravenous fluid (IVF) administration in critically ill children. Previous efforts have described opportunities to reduce positive cumulative fluid balance (CFB) in this population but have not been widely implemented. In the wake of Hurricane Helene, a national IVF shortage led to the implementation of IVF conservation guidelines. We sought to determine if this was associated with a reduction in IVF use and CFB.

**Methods:**

The present study is a four-site cohort study of critically ill children utilizing a federated data collection framework to extract patient age, sex, weight, and daily fluid intake/output for days 1–4 of all admissions 28 days prior to and 28 days after the implementation of IVF conservation guidelines. Guidelines were individualized per institution. Total fluid intake, total IVF intake, % intake from IVF, and % CFB were compared between pre- and post-IVF conservation groups.

**Results:**

All sites had similar conservation recommendations. There were 633 patients admitted pre- and 619 patients admitted post-IVF conservation guideline implementation, with similar age and weight distributions. There was no significant difference in IVF use pre- and post-IVF conservation; 29–35% of patients had > 5% CFB on day 1 pre-IVF conservation while 27–39% did post-conservation, with increasing numbers on day 2.

**Conclusions:**

Even in the setting of a national IVF shortage, simple recommendations without structured change were insufficient to change IVF administration practices. This indicates additional practices will be needed to reduce IVF intake and % CFB in this vulnerable population.

**Graphical abstract:**

**Figure:**
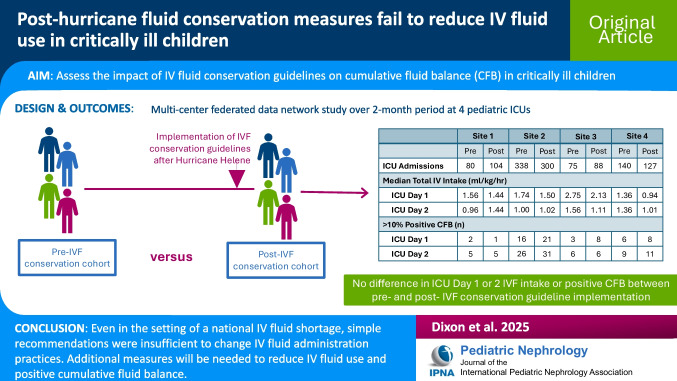
A higher resolution version of the Graphical abstract is available as Supplementary information

**Supplementary Information:**

The online version contains supplementary material available at 10.1007/s00467-025-06931-x.

## Introduction

Critically ill children in the pediatric intensive care unit (PICU) receive intravenous fluids (IVF) for resuscitation, maintenance hydration (often calculated using the Holliday–Segar equation), and medication delivery [1]. While IVF administration is often necessary, there are risks associated with excessive fluid administration. Positive cumulative fluid balance (CFB) is associated with higher mortality and longer PICU length of stay (LOS) [2, 3]. Recent efforts have sought to evaluate the amount of IVF prescribed to PICU patients and find opportunities for improvement [4, 5].

On September 29, 2024, Hurricane Helene caused catastrophic damage and flooding in western North Carolina, near the plant of a major IVF producer. The resulting halt in production and distribution led to an acute national IVF shortage, stressing hospital systems and leading to a call for providers to more carefully consider IVF use [6].

Our existing multicenter collaborative has been studying ways to reduce CFB in critically ill children. In response to this crisis, each of our academic PICUs implemented IVF conservation guidelines. The objective of this study was to identify associations between fluid conservation guidelines and CFB. We conducted a multicenter retrospective cohort study of IVF administration practices 1 month prior to and 1 month after the initiation of IVF conservation initiatives. We hypothesized that hospital-wide mandates for IVF conservation would result in less IVF administered to PICU patients, and that we would observe variability among hospitals with different conservation policies.

## Methods

### Study design

This was a retrospective observational study including patients admitted to the PICU at four tertiary care centers. The institutional review boards (IRB) at each site (New York University Langone Health, Children’s Hospital of Philadelphia, University of Alabama at Birmingham, and University of Rochester Medical Center) deemed this study exempt from IRB review. This study was conducted in accordance with the ethical standards of the Helsinki Declaration of 1975. Sites 1 and 2 included patients admitted to either the PICU or the cardiac intensive care unit (CICU), while sites 3 and 4 included only PICU admissions. All sites identified the date that IVF conservation strategies were implemented in their ICU (“implementation date”) and described their approaches to IVF conservation. We utilized a federated data collection framework to collect and aggregate clinical data without sharing row-level data among sites [7].

We compared all ICU admissions in the month prior to each site’s identified implementation date (“pre”) to the month following this date (“post”). All patients admitted during these time periods were included. We included a 4-day washout period surrounding the implementation date. For each ICU encounter, we recorded patient demographics (age, admission weight), fluid intake (amount, route), urine output and occurrences (output that occurred but could not be quantified), and LOS from the electronic health record (EHR). We excluded from CFB analysis all ICU encounters with urine occurrence counts on or prior to that day to not exaggerate CFB values. Each site extracted their own data and ran identical analysis scripts.

#### Analysis

Consistent with prior studies [2], we defined day 0 of ICU stay as admission time through 7 AM the following day. We defined day 1 as the subsequent 24 h (7 to 7 AM), day 2 as the following 24 h, and so on. Total intake and IVF intake were standardized to ml/kg/h. We calculated fluid metrics for days 1–4, including the following: percent of total intake as IVF as total IVF intake/(total enteral + IVF intake), percent of Holliday–Segar received as total IVF/(4 × first 10 kgweight + 2 × number of kg between 10 and 20 + 1 × number of kg > 20) [1], and percent CFB as (cumulative fluid in [L]––cumulative fluid out [L])/PICU admission weight (kg) × 100 [8]. Day 0 fluid metrics were not included in the analysis, given that this could represent a variable number of hours depending on the time of admission. Day 0 is also a period of active fluid resuscitation, and not the target of fluid conservation guidelines. Differences between groups were compared using the chi-square test of independence, Fisher’s exact test, or Wilcoxon rank sum as appropriate using analysis scripts written in R (https://www.r-project.org). *p *values of < 0.05 were considered significant.

## Results

### Fluid conservation recommendations

**Fig. 1:**
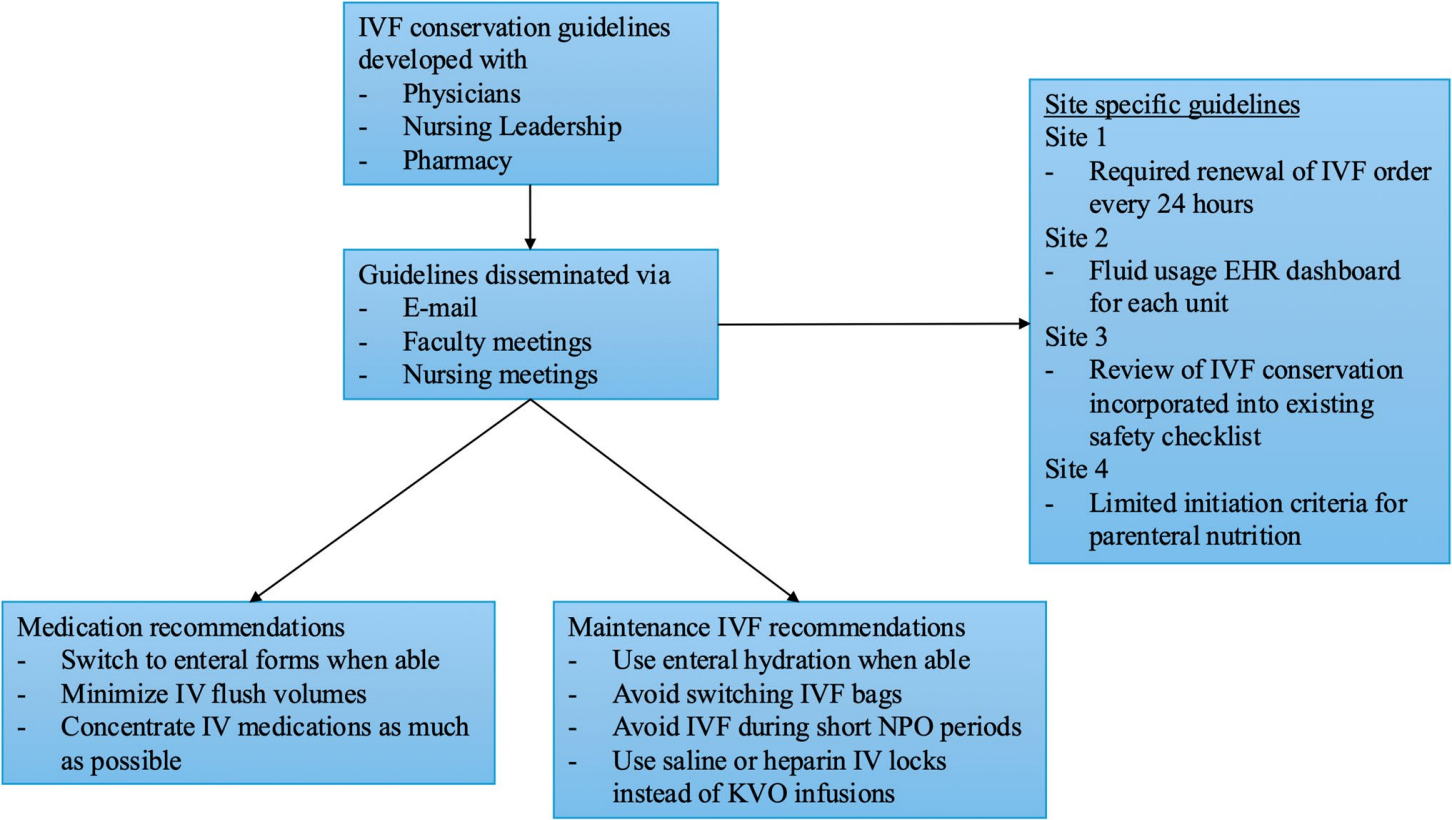
Common themes of fluid conservation guidelines

All sites had similar recommendations regarding IVF conservation practices (Fig. 1). Guidelines were created with input from physician, nursing, and pharmacy staff, and materials were distributed via email and in-person meetings. Common themes identified were using enteral hydration when possible, converting medications to enteral forms, limiting use of fluids for short nil per os (NPO) times, and avoiding switching bags of fluid once started. No site required specific practice changes or utilized interruptive EHR alerts.

### Fluid measurements pre- and post-conservation

Cohort demographics are shown in Table 1. There were 633 pre-encounters and 619 post-encounters, with sites 1 and 3 having more patients post and sites 2 and 4 having fewer. There was an expected decrease in the number of encounters between days 1 to 2 (Supplementary Table 1). Most sites had a slight male predominance, with sites 3 and 4 demonstrating a significant association between sex and temporal grouping. All sites had more younger (< 1 year) and smaller (< 10 kg) patients in the post-group, although these differences were not significant. The rate of mechanical ventilation was not different between pre- and post-groups at any site. There was also no significant difference in the number of patients with LOS > 7 days or difference in overall mortality at any site.

**Table 1:** Description of the cohort

	**Site 1**		**Site 2**		**Site 3**		**Site 4 **	
	Pre	Post	Pre	Post	Pre	Post	Pre	Post
ICU admissions, *n*	80	104	338	300	75	88	140	127
Female, *n* (%)	45 (56%)	50 (48%)	140 (41%)	130 (43%)	40 (53%)	33 (38%)	72 (51%)	49 (39%)
Age, *n* (%)								
< 1 year	13 (16%)	29 (28%)	75 (22%)	81 (27%)	11 (15%)	12 (14%)	21 (15%)	30 (24%)
≥ 1 and < 4 years	19 (24%)	25 (24%)	82 (24%)	67 (22%)	23 (31%)	35 (40%)	35 (25%)	28 (22%)
≥ 4 and < 12 years	22 (28%)	31 (30%)	97 (29%)	88 (29%)	24 (32%)	25 (28%)	40 (29%)	26 (21%)
≥ 12 years	26 (33%)	19 (18%)	84 (25%)	64 (21%)	17 (23%)	16 (18%)	44 (31%)	43 (34%)
Weight, *n* (%)								
< 10 kg	14 (18%)	29 (28%)	85 (25%)	84 (28%)	10 (13%)	17 (19%)	28 (20%)	34 (27%)
10–30 kg	34 (43%)	44 (42%)	143 (42%)	129 (43%)	40 (53%)	47 (53%)	51 (36%)	40 (32%)
≥ 30 kg	32 (40%)	31 (30%)	110 (33%)	87 (29%)	25 (33%)	24 (27%)	61 (44%)	53 (42%)
Race, *n* (%)^a^								
White or Caucasian	49 (61%)	55 (53%)	129 (38%)	121 (40%)	41 (55%)	47 (54%)	65 (46%)	63 (50%)
Black or African American	17 (21%)	21 (20%)	100 (30%)	87 (29%)	8 (11%)	18 (20%)	54 (39%)	42 (33%)
Asian	2 (3%)	4 (4%)	18 (5%)	6 (2%)	8 (11%)	7 (8%)	0	1 (< 1%)
Other or multiple responses	11 (14%)	18 (17%)	70 (21%)	63 (21%)	7 (9%)	5 (6%)	17 (12%)	15 (12%)
Unknown or refused	1 (1%)	6 (6%)	21 (6%)	20 (7%)	11 (15%)	11 (13%)	4 (3%)	6 (5%)
Ethnicity, *n* (%)								
Not Hispanic or Latino	64 (80%)	76 (73%)	262 (78%)	231 (77%)	34 (45%)	38 (43%)	124 (89%)	111 (87%)
Hispanic or Latino	8 (10%)	7 (7%)	61 (18%)	49 (16%)	10 (13%)	9 (10%)	15 (11%)	11 (9%)
Other, unknown, or refused	8 (10%)	21 (20%)	15 (4%)	20 (7%)	31 (41%)	41 (47%)	1 (< 1%)	5 (4%)
Patients LOS > 7 days, *n* (%)	15 (19%)	20 (19%)	51 (15%)	46 (15%)	12 (16%)	10 (11%)	11 (8%)	11 (9%)
Mechanical ventilation, *n* (%)	29 (36%)	37 (36%)	79 (23%)	86 (29%)	25 (33%)	24 (27%)	40 (29%)	38 (30%)
Hospital disposition								
Home or self-care	69 (88%)	92 (94%)	304 (93%)	250 (88%)	59 (86%)	71 (91%)	120 (92%)	113 (93%)
Another facility or other	8 (10%)	4 (4%)	16 (5%)	28 (10%)	7 (10%)	4 (5%)	2 (2%)	3 (2%)
Death	1 (1%)	2 (2%)	7 (2%)	5 (2%)	3 (4%)	3 (4%)	8 (6%)	5 (4%)

^a^Centers for Disease Control and Prevention (CDC) definitions used; “Native Hawaiian or Pacific Islander” and “American Indian or Alaskan Native” collapsed into “other or multiple responses” given** ≤ **1 encounter in all groups

Days 1 and 2 fluid metrics are shown in Table 2. Overall, IVF volumes on days 1 and 2 of ICU admission were not consistently different between pre- and post-groups at any site. Total fluid intake was either the same or slightly higher in the post-group at all sites on both days, though this only reached significance at site 4 on day 2. Total IVF intake was lower in the post-group at most sites; however, this difference was also not significant. The percent of total intake as IVF was variable across sites and decreased from pre to post. Although this decrease was only significant on day 1 at site 1 (*p* = 0.04) (Table 2), sites 3 and 4 showed a similar but nonsignificant trend on both days 1 and 2 (*p* < 0.1). There was no significant difference in the number of patients with 5% or 10% positive CFB at any site on either day 1 or 2. Across all sites, 29–35% of pre-patients and 27–39% of post-patients had ≥ 5% CFB on day 1, with higher percentages of patients on day 2 (33–54% pre and 42–52% post). A smaller percentage of patients reached ≥ 10% CFB, with a similar increase from day 1 to day 2 in both pre- and post-groups.

**Table 2:** Fluid metrics

	Site 1		Site 2		Site 3		Site 4	
	Pre	Post	Pre	Post	Pre	Post	Pre	Post
ICU admissions (*n*)	80	104	338	300	75	88	140	127
Total intake (IV + enteral) (ml/kg/h, [IQR])^a^								
ICU day 1	2.45 [1.48, 4.25]	3.27 [2.26, 4.28]	3.34 [2.11, 4.44]	3.30 [1.97, 4.65]	3.34 [2.14, 4.26]	3.60 [2.53, 4.28]	2.69 [1.35, 4.38]	2.82 [1.28, 5.01]
ICU day 2	2.72 [1.24, 4.72]	3.35 [2.22, 4.30]	3.02 [1.97, 4.45]	3.21 [1.74, 5.27]	3.25 [2.21, 4.30]	3.37 [1.89, 4.33]	2.65 [1.62, 4.09]**	3.51 [2.02, 5.54]**
Total IV intake (ml/kg/h, [IQR])^a^								
ICU day 1	1.56 [0.63, 3.30]	1.44 [0.09, 3.26]	1.74 [0.76, 2.82]	1.50 [0.28, 3.18]	2.75 [1.19, 3.91]	2.13 [0.66, 3.70]	1.36 [0.37, 2.52]	0.94 [0.12, 2.97]
ICU day 2	0.96 [0.24, 2.83]	1.44 [0.00, 3.16]	1.00 [0.08, 2.45]	1.02 [0.03, 2.65]	1.56 [0.61, 3.38]	1.11 [0.02, 3.34]	1.36 [0.15, 2.58]	1.01 [0.02, 2.76]
Percent of total intake as IVF (%, [IQR])^a^								
ICU day 1	89.2 [45.5, 100.0]**	64.9 [6.3, 100.0]**	59.4 [25.7, 97.0]	53.3 [15.1, 96.6]	93.1 [64.0, 100.0]*	76.9 [29.4, 100.0]*	70.6 [26.7, 100.0]*	58.9 [9.5, 95.4]*
ICU day 2	70.4 [17.2, 95.7]	62.0 [0.0, 100.0]	32.4 [4.1, 84.3]	32.0 [3.3, 80.1]	71.8 [17.7, 98.7]*	42.1 [2.54, 85.8]*	59.2 [10.9, 93.1]*	40.5 [0.65, 80.2]*
Percent of calculated Holliday–Segar received (%, [IQR])^a^								
ICU day 1	71.3 [27.2, 105.3]	57.2 [2.5, 102.9]	62.3 [25.2, 101.9]*	50.3 [12.7, 103.1]*	93.0 [37.6, 134.7]	82.5 [25.2, 120.5]	57.2 [17.6, 106.9]	44.5 [4.2, 114.2]
ICU day 2	39.3 [8.2, 89.5]	45.1 [0.0, 96.7]	35.3 [3.1, 90.4]	33.9 [1.6, 94.1]	71.3 [19.7, 93.2]	37.5 [0.6, 102.2]	62.4 [8.8, 96.9]	35.5 [0.5, 117.1]
> 5% positive CFB (*n*)^b^								
ICU day 1	11 (30%)	14 (29%)	78 (31%)	74 (34%)	20 (35%)	25 (39%)	32 (29%)	23 (27%)
ICU day 2	5 (33%)	13 (52%)	82 (45%)	67 (42%)	19 (54%)	22 (48%)	24 (39%)	25 (50%)
> 10% positive CFB (*n*)^b^								
ICU day 1	2 (5%)	1 (2%)	16 (6%)	21 (10%)	3 (5%)	8 (13%)	6 (6%)	8 (9%)
ICU day 2	5 (33%)	5 (20%)	26 (14%)	31 (20%)	6 (17%)	6 (13%)	9 (15%)	11 (22%)

*p ≤ 0.1; **p ≤ 0.05

^a^Patients with 0 ml intake excluded from this analysis

^b^Patients with urine occurrences (volume not recorded) excluded from this analysis

In supplementary analysis, the same metrics were compared for days 3 and day 4. Sites 3 and 4 had a significant decrease in percent of total intake as IVF on day 4. No significant differences were observed for any other metric (Supplementary Table 2).

## Discussion

In this four-site study of fluid practices surrounding Hurricane Helene, there was no significant difference in IVF administration or CFB between pre- and post-fluid conservation implementation. Despite nation- and hospital-wide mandates and the development of targeted guidelines, there was no observed change in clinical practice. It is not surprising that we also found no difference in the prevalence of positive CFB in the post-groups.

These findings were consistent across PICUs of different sizes and hospitals in small and large metropolitan areas. Overall CFB > 5% and > 10% on days 1 and 2 were very similar to percentages reported at the same sites over 6-month periods just 1.5 years prior [9]. It is worth noting that some sites had a decrease in the percentage of total intake given as IVF, while total intake increased. This implies that, rather than a reduction in IVF use, there was an increase in enteral fluid administration. There was a trend toward decreased IVF intake at sites 3 and 4, although this was not significant.

It is likely that the reason for these null findings is not because changes were not attempted, but rather that more significant interventions are required to limit CFB in critically ill children. Practice guidelines and recommendations related to passive fluid restriction, even within the context of a national IVF shortage, were insufficient to change the amount of IVF administered. However, other groups have shown in single-center prospective quality improvement studies that improved recognition of positive CFB and mandated decrease in maintenance IVF rates can reduce CFB [4, 10]. Our study indicates that guidelines to limit IVF use alone do not significantly impact the amount of IVF given and therefore do not reduce the burden of positive CFB in this population.

While sites had varying degrees of detail in their recommendations for IVF conservation, all had similar themes of using enteral medications and hydration where possible, minimizing IV flush volumes and vein patency infusions, and avoiding IVF use for short NPO periods. All sites had different mechanisms for encouraging adherence to recommended conservation guidelines. Site 1 required IVF to be re-ordered every 24 h, site 2 utilized a fluid dashboard that tracked unit-specific fluid use, site 3 utilized a pre-existing safety checklist, and site 4 implemented more restrictive criteria for initiation of total parenteral nutrition (TPN). The variability in fluid conservation guidelines reflects the lack of standardization across hospitals during this period. Ultimately, fluid management decisions remained entirely with the ICU team without any “hard stops” for prescribing or administering fluids. This may have contributed to the lack of change observed.

There are limitations to this study. We did not collect data on type of fluid prescribed (e.g., balanced vs. unbalanced crystalloids), why they were prescribed (maintenance vs. carriers vs. medications), or frequency of fluid bag changes. All sites did have recommendations to limit changing fluid type prior to finishing the bag, which may have reduced overall hospital IVF usage but not individual patient exposure. We also did not collect diagnostic codes or severity of illness measures, so it is possible that a discrepancy between pre- and post-groups could have accounted for some of the observed fluid administration variation. We believe this to be less likely given ICU LOS, mechanical ventilation, and mortality did not differ, and the CFB we observed was similar not only pre- and post, but also to historical data from the same sites. We could not account for insensible losses, which may have affected CFB. Accurate daily weights could reflect CFB with insensible losses; however, weight was not measured consistently enough at any site to use in this way [9]. Finally, there was likely variability between sites for implementation of guidelines and adherence by clinical teams, which may have minimized the observed effect.

Future prospective studies aimed at reducing CFB in the PICU should focus on targeted, prescriptive interventions rather than practice guidelines used in this time period. Necessary balancing measures, including markers of kidney function and end-organ perfusion, should also be tracked. This type of study could allow for monitoring of adherence across sites, show the impact of more conservative fluid management in critically ill children, and impact future IVF practices.

## Conclusion

Despite a national IVF shortage and specific IVF conservation guidelines, no significant difference in IVF use was observed in this time period among a multicenter collaborative focused on mitigating fluid overload in critically ill children. Further investigation is required to understand IVF prescribing practices and barriers to reduce IVF volume in critically ill children.

## Supplementary information

Below is the link to the electronic supplementary material.

Graphical abstract (PPTX 73.6 KB)

## Data Availability

The data that support the findings of this study are not openly available due to reasons of sensitivity and may be made available upon reasonable request. Investigators’ proposed use of data must be approved by an independent review committee, and analysis must directly address the proposed aims. Requests should be directed to Adam C. Dziorny (adam_dziorny@urmc.rochester.edu). This manuscript has been posted to a pre-print server and can be found here: 10.1101/2025.02.04.25321427v1.
