# Supplementary material for: Post-hurricane fluid conservation measures fail to reduce IV fluid use in critically ill children
Source: Pediatr Nephrol. 2025 Aug 19;40(12):3825–30. doi: 10.1007/s00467-025-06931-x (PMC12549755; doi:10.1007/s00467-025-06931-x)
Supplement: Supplementary file 1 — Graphical abstract (PPTX 73.6 KB) [file 467_2025_6931_MOESM1_ESM.pptx]

## Slide 1
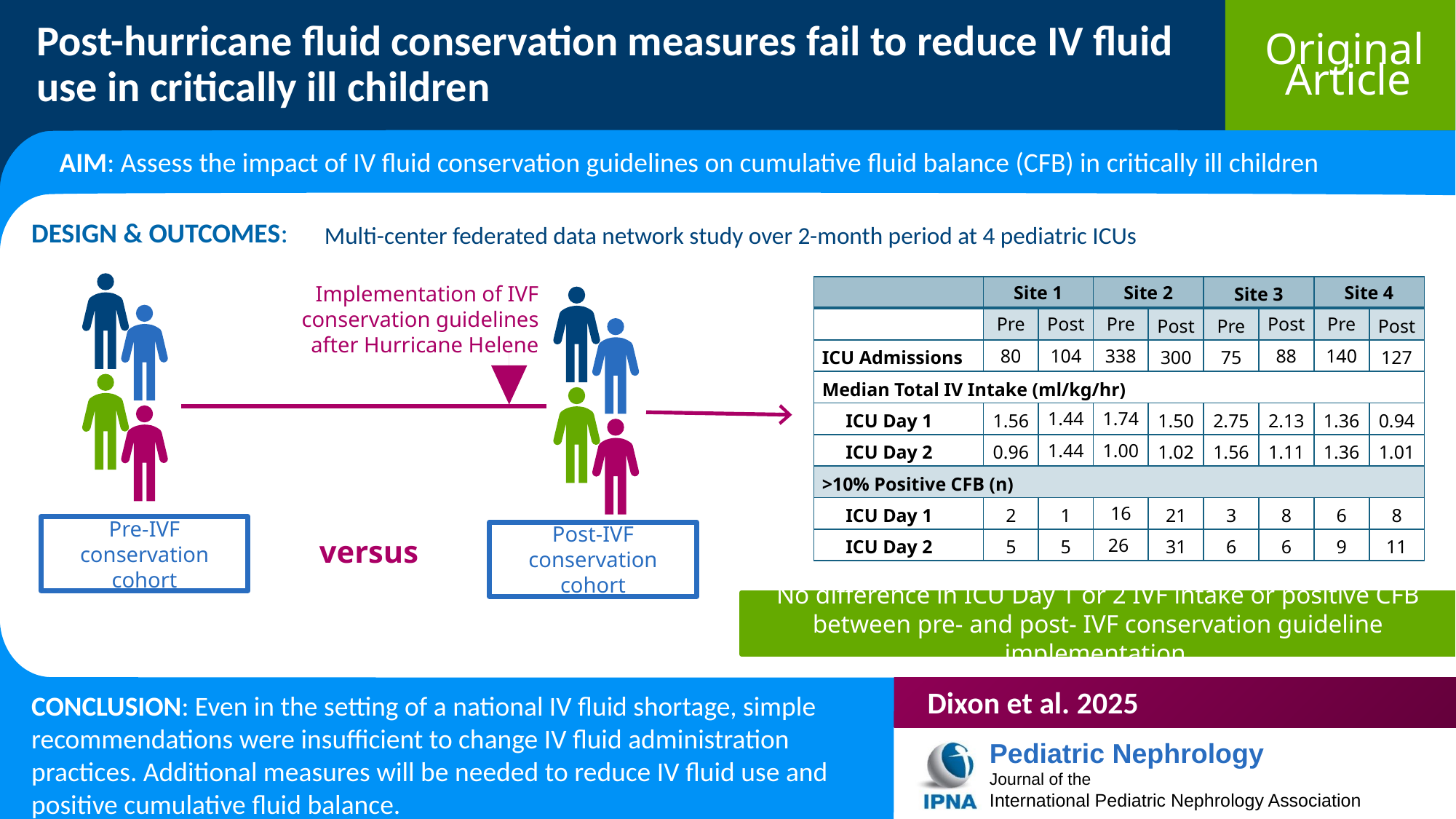

Post-hurricane fluid conservation measures fail to reduce IV fluid use in critically ill children
AIM: Assess the impact of IV fluid conservation guidelines on cumulative fluid balance (CFB) in critically ill children
DESIGN & OUTCOMES:
Multi-center federated data network study over 2-month period at 4 pediatric ICUs
Implementation of IVF conservation guidelines after Hurricane Helene
Pre-IVF conservation cohort
Post-IVF conservation cohort
versus
| | Site 1 | | Site 2 | Site 2 | Site 3 | | Site 4 | Site 4 |
| --- | --- | --- | --- | --- | --- | --- | --- | --- |
| | Pre | Post | Pre | Post | Pre | Post | Pre | Post |
| ICU Admissions | 80 | 104 | 338 | 300 | 75 | 88 | 140 | 127 |
| Median Total IV Intake (ml/kg/hr) | | | | | | | | |
| ICU Day 1 | 1.56 | 1.44 | 1.74 | 1.50 | 2.75 | 2.13 | 1.36 | 0.94 |
| ICU Day 2 | 0.96 | 1.44 | 1.00 | 1.02 | 1.56 | 1.11 | 1.36 | 1.01 |
| >10% Positive CFB (n) | | | | | | | | |
| ICU Day 1 | 2 | 1 | 16 | 21 | 3 | 8 | 6 | 8 |
| ICU Day 2 | 5 | 5 | 26 | 31 | 6 | 6 | 9 | 11 |
No difference in ICU Day 1 or 2 IVF intake or positive CFB between pre- and post- IVF conservation guideline implementation
Dixon et al. 2025
CONCLUSION: Even in the setting of a national IV fluid shortage, simple recommendations were insufficient to change IV fluid administration practices. Additional measures will be needed to reduce IV fluid use and positive cumulative fluid balance.
